# A systematic review investigating emerging trends between Extreme Weather Events (EWEs) and infectious disease outbreaks in South Africa

**DOI:** 10.3389/fpubh.2026.1778784

**Published:** 2026-03-16

**Authors:** Natalie Dickinson, Llinos Haf Spencer, Caroline Miller, Nisha Nadesan-Reddy, Serestina Viriri, Muhammad Zeeshan Shakir, Michael Gebreslasie, David Ndzi, Ozayr Haroon Mahomed, Saloshni Naidoo, Mary Lynch, Fiona Luisa Henriquez

**Affiliations:** 1 School of Health and Life Sciences, University of the West of Scotland, Paisley, United Kingdom; 2 Faculty of Nursing and Midwifery, Royal College of Surgeons in Ireland, Dublin, Ireland; 3 Faculty of Life Sciences and Education, University of South Wales, Cardiff, United Kingdom; 4 Discipline of Public Health, Howard College, University of Kwazulu-Natal, Durban, South Africa; 5 School of Mathematics, Statistics, and Computer Science, University of Kwazulu-Natal, Durban, South Africa; 6 School of Computing, Engineering and Physical Sciences, University of the West of Scotland, Paisley, United Kingdom; 7 School of Mathematics, Statistics, and Computer Science, University of Kwazulu-Natal, Durban, South Africa; 8 School of Electrical and Mechanical Engineering, University of Portsmouth, Portsmouth, United Kingdom; 9 Department of Civil and Environmental Engineering, University of Strathclyde, Glasgow, United Kingdom

**Keywords:** community health, drought, early warning system, extreme weather events, floods, health system resilience, South Africa, pathogens

## Abstract

**Introduction:**

Extreme weather events (EWEs) are increasing in frequency and intensity due to climate change, exacerbating health risks, particularly for vulnerable populations. Infectious diseases are climate-sensitive, yet microbial dynamics during and after EWEs remain poorly understood. This systematic review examines if there are emerging trends and associations in infectious disease outbreaks and health care following EWEs in South Africa.

**Methods:**

A comprehensive search across fifteen electronic databases was conducted using Cochrane systematic review principles, including double screening and double extraction. Studies describing outbreaks of infectious diseases related to EWEs in South Africa were included, considering all study designs. A PRISMA diagram details the screening process, and quality appraisal was conducted using JBI checklist tools or a mixed-methods assessment tool.

**Results:**

The review did not identify any primary studies that explicitly examined the presence of specific pathogens contaminating the environment in relation to EWEs in South Africa. Instead, the available literature predominantly focused on clinical symptom patterns or broader syndromic descriptions, with limited attention to pathogen-specific detection or characterisation. This highlights a significant evidence gap in microbial-level assessments of how EWEs influence environmental contamination. However, findings indicate the presence of infectious diseases post-EWEs, with three key themes emerging: (1) climate variables, (2) population vulnerabilities, and (3) policy effects. The review highlights the need for longitudinal microbiological data to improve outbreak prediction and preparedness.

**Discussion:**

This review underscores a significant research gap and calls for an integrated approach combining environmental monitoring with pathogen diagnostics. Large-scale longitudinal studies and enhanced collaboration public health, environmental surveillance, laboratory capacity, and disaster preparedness are needed. Early Warning Systems should incorporate climate variables to predict disease outbreaks, addressing the limited diagnostics, lack of environmental microbiology, fragmented surveillance data effectively.

## Background

The surge in extreme weather events (EWEs) over the past 25 years has resulted in the most devastating impacts, in terms of health status, economic losses, displacement, infrastructure damage. During the period 2000–2019, six of the ten countries most affected by EWEs were located on the African continent (1). In South Africa, the frequency and intensity of EWEs, including droughts, floods, and heatwaves, have increased due to climate change (2) with widespread regional and localized variability (3, 4). The coastal region of KwaZulu-Natal (KZN) is experiencing a gradual increase in temperatures and in the frequency of extreme rainfall days. These intense rainfall events now contribute a greater proportion of annual rainfall than sustained wet periods, increasing the risk of drought between events and leaving dry soils more susceptible to flash flooding and surface runoff during heavy rain (4). This significantly influences public health, particularly the spread of infectious diseases, as evidenced in global studies (5–7). South Africa's diverse climate zones, coupled with socio-economic disparities, multidimensional inequalities and limited healthcare infrastructure in certain regions create an environment where climate-induced disease outbreaks pose a critical challenge (8–11). In general infections, such as malaria, cholera, and diarrheal diseases, are closely linked to environmental changes, exacerbated by heavy rainfall, flooding, and droughts (6). Other geographical regions around the world, for example the continent of Asia often experiences flood-related cholera outbreaks, particularly in regions with poor sanitation and overcrowding (12). In Pakistan, extreme monsoon floods in 2022, led to a significant rise in waterborne diseases, including hepatitis E and acute gastroenteritis, as millions were displaced and forced to consume contaminated water (13). In South America, Peru and Brazil have also witnessed a resurgence of vector- and waterborne diseases, including dengue fever, leptospirosis and malaria, in the wake of El Niño-driven flooding events (14, 15).

On the African continent, increased flooding and heavy rainfall often lead to the contamination of water supplies, fuelling outbreaks of cholera, typhoid fever, and other diarrheal diseases (16, 17). During the 2018 cholera outbreak in Zimbabwe, prolonged rainfall overwhelmed sanitation infrastructure, resulting in a rapid spread of *Vibrio cholerae* (18). Similarly, in Mozambique, Cyclone Idai (2019) led to massive flooding, triggering a large-scale cholera epidemic (19). The Durban floods of 2012 and 2022 had severe consequences for human health leading to widespread water contamination, increasing cases of cholera, diarrhea, and leptospirosis (20). The April 2022 floods, regarded as one of the deadliest climate disasters in South Africa's history, resulted in over 435 fatalities and displaced thousands, exacerbating vulnerabilities in informal settlements (21). The destruction of sanitation infrastructure and potable water supplies created ideal conditions for waterborne diseases, including typhoid fever and *E. coli* infections (22). Additionally, stagnant water left in the aftermath facilitated mosquito breeding, heightening the risk of vector-borne diseases such as malaria and dengue fever (23). The combined effects of displacement, overcrowding in emergency shelters, and inadequate access to healthcare services further intensified the spread of respiratory infections and other communicable diseases (24). Conversely, drought conditions threaten water security, often forcing vulnerable populations to rely on unsafe drinking water sources, increasing the risk of gastrointestinal infections and waterborne illnesses (25, 26).

In East Africa, severe droughts have reduced access to potable water, leading to a spike in diarrheal diseases and acute malnutrition (27). Prolonged droughts have been linked to increased concentrations of harmful algal blooms, contributing to toxic cyanobacteria outbreaks, which pose a threat to both human and animal health (28). The link between environmental conditions and infectious disease outbreaks has garnered increasing attention in global health research, especially in regions that are both highly vulnerable to climate change and predisposed to public health crises (5, 9, 29). In South Africa, there is already a higher level of *E. coli* in the surface water system than expected due to inadequate water treatment (30). The level of *E. coli* is likely to rise during EWEs through water contamination.

The specific impact of EWEs on pathogen dynamics, particularly in relation to emerging and re-emerging infectious diseases, remains poorly understood (31). South Africa's recent experiences with recurring droughts and floods, particularly in marginalized and rural communities, underscore the urgent need to understand how climate change-driven EWEs influence infectious disease outbreaks (32, 33). To our knowledge, no previous systematic review has comprehensively examined the intersection of climate change, EWEs, and infectious disease outbreaks in South Africa.

### Aim

The aim of this systematic review is to understand emerging trends in disease outbreaks following EWEs in South Africa.

Research questions:

1. What is the incidence of infection and what pathogens are causing outbreak(s)?

2. Who are the vulnerable and affected individuals, and do they have common characteristics (age, sex, underlying conditions, property ownership)?

3. What are the impacts of EWEs such as flooding on policy decision making at local and national government levels?

## Methods

This review addressed the research question registered on PROSPERO (34): “What are the emerging trends in EWEs and infectious disease outbreaks in South Africa?”

### Search strategy

A comprehensive search strategy was developed using appropriate key words, free text terms and Boolean operators to maximize the retrieval of potentially relevant studies (34). Full search terms can be found in Supplementary File 1. The key words included: “South Africa“ AND (“Extreme Weather Events” OR *alternate terms*) AND (“infectious disease” OR *alternate terms, including disease and pathogen names*). The search was conducted across various electronic databases (Web of Science, PubMed, Science Direct, EBSCO (12 databases) and Cochrane Library) for studies published between January 2014 – June 2024 to capture contemporary evidence reflecting recent climate variability, advances in disease surveillance systems, and policy developments in South Africa, including the National Climate Change Response Policy and subsequent adaptation strategies. This timeframe ensures relevance to current governance and health system contexts. as there has been a marked increase in the frequency, intensity, and impact of EWEs in South Africa, including severe droughts, floods, heatwaves, and storms in the past decade.

### Eligibility criteria

Eligibility was based on the following inclusion and exclusion criteria.

#### Inclusion criteria

The inclusion criteria encompass literature with a focus on EWEs and infectious diseases occurring in South Africa. Eligible sources include peer-reviewed journal articles, government and non-governmental organization reports, and academic dissertations. A wide range of study designs is considered, including qualitative, quantitative, and mixed-methods studies, as well as systematic reviews, scoping reviews, meta-analyses, and rapid reviews, where data extraction specific to South Africa is possible.

#### Exclusion criteria

We excluded articles not published in English due to limited translation resources. We also excluded studies that did not specifically address EWEs, such as heatwaves, floods, hurricanes, droughts, and wildfires, and their association with infectious diseases, including cholera, dengue, malaria, leptospirosis, and respiratory infections.

### Screening and study selection

Screening and eligibility determination used a two-reviewer system (with consensus for disagreements and conferral with a third-party adjudicator if a consensus was unable to be reached) on the Rayyan systematic review management system. Studies were identified and screened by reviewing the title and abstract to remove all articles that clearly did not meet the eligibility criteria, followed by full text screening by two independent reviewers. Details about the included and excluded studies are presented in a PRISMA diagram (35, 36). Systematic reviews were included where unique data was extractable in accordance with published guidance (37, 38).

### Data extraction

Four reviewers were involved in data extraction (ND, FH, LHS, ML). The included articles were all reviewed and critically appraised independently by two reviewers, and data added to a data extraction table (see Supplementary File 2). Disagreements were resolved by a third independent reviewer.

### Methodological quality assessment

All studies were rated for quality using study-specific quality appraisal tools from the Joanna Briggs institute (39–41). The authors adopted a scoring system to rate quality as high, medium or low (42). See Supplementary File 2 for the quality appraisal tables.

### Data synthesis

Given the heterogeneity in outcome definitions across included studies, key outcome terms were operationalised to ensure consistency of interpretation. “Outbreak” was defined as either (i) a formally declared outbreak reported by public health authorities, or (ii) a statistically identified excess above expected baseline levels where explicitly described by study authors. No included study constituted a formally investigated outbreak with microbiological confirmation.

“Incidence” was defined as reported case counts or rates derived from routine surveillance systems, hospital admissions, or public health reporting platforms, depending on the primary data source. In several studies, incidence reflected aggregated admission or surveillance data rather than laboratory-confirmed case registries.

“Pathogen attribution” was classified according to level of diagnostic specificity: (i) laboratory-confirmed pathogen outcomes; (ii) clinically defined infectious outcomes based on routine diagnostic coding; (iii) syndromic outcomes defined by symptom categories; and (iv) proxy indicators such as medication sales. This framework enabled structured comparison across diverse study designs and prevented overinterpretation of pathogen-specific relationships.

This synthesis was conducted in line with Cochrane principles for analysis and reporting, ensuring a systematic, transparent, and reproducible approach to evidence integration. Findings were grouped thematically to capture patterns related to types of EWEs, associated pathogens or disease outcomes, environmental and socio-economic drivers, and surveillance or response mechanisms. This approach accommodated the heterogeneity of the study designs, data sources, and outcome measures.

## Results

**Figure 1:**
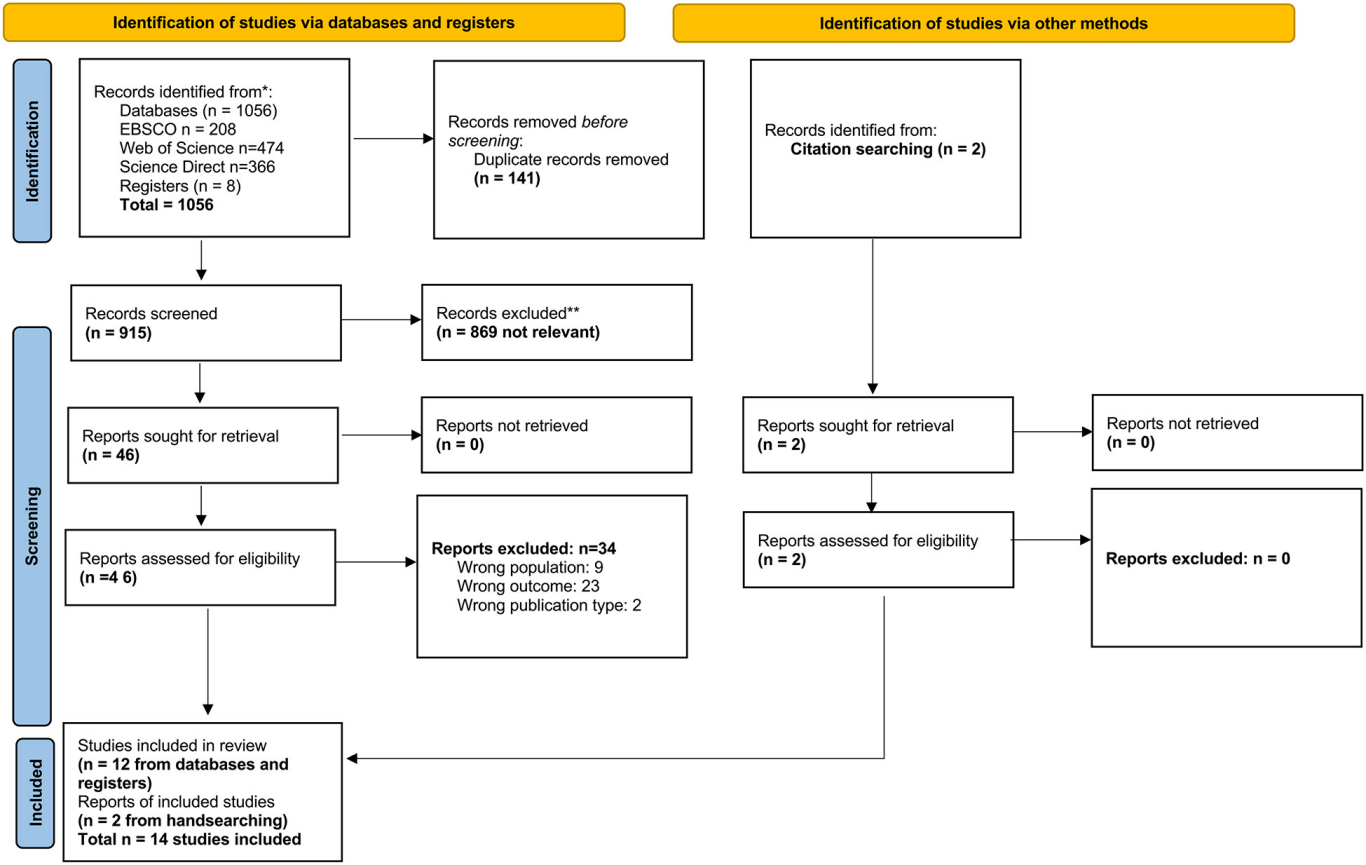
PRISMA diagram illustrating the process of identification, screening and inclusion of studies. *See Supplementary file 1, **through two reviewer screening.

The PRISMA diagram (Figure 1) visually represents the process of identifying, screening and including studies in this review (36).

Fourteen studies were included in the systematic review and classified into types of study (Supplementary Figure S1). The included studies were categorized according to (i) mode of infectious disease transmission (waterborne/enteric, vector-borne, respiratory), and (ii) level of outcome ascertainment (laboratory-confirmed pathogen data, clinically defined admissions/incidence data, syndromic outcomes, or proxy indicators such as pharmaceutical sales). This classification framework is summarized in Table 1 and guided subsequent thematic synthesis. Table 1 presents summary data (e.g., study design, location, climate variables, outcomes) of the included studies. The full data extraction and critical appraisal information can be found in Supplementary File 2. All studies considered the effects of rainfall and/or temperature, apart from Amegah et al.'s (43) systematic review, which considered temperature only.

**Table 1:** Characteristics of included studies classified by transmission mode and level of outcome ascertainment.

**Study**	**Design**	**Province/region**	**Climate variables**	**Transmission category**	**Infectious outcomes**	**Level of outcome data**	**Data source type**
Ikeda et al. (44)	Quantitative (correlational)	Limpopo	Temperature, Rainfall	Vector-borne	Malaria	Clinical incidence (surveillance)	Aggregated malaria case records
Ikeda et al. (51)	Quantitative (correlational)	Limpopo	Temperature, Rainfall	Waterborne/Enteric	Diarrhoeal disease	Clinical admissions	Hospital admission records
Kapwata et al. (45)	Quantitative (time-series)	Limpopo	Temperature, Rainfall	Vector-borne; Respiratory; Enteric	Malaria, Pneumonia, Diarrhea	Clinical admissions	Hospital records
Kapwata et al. (65)	Mixed-methods	Limpopo	Temperature, Rainfall	Waterborne/Enteric	Bacterial enteropathogens	Laboratory-confirmed	NHLS specimen data
Lee et al. (61)	Mixed-methods	Western Cape	Temperature, Rainfall	Waterborne/Enteric	Diarrhoeal disease	Clinical surveillance	Public health system data
Abdullahi et al. (63)	Modeling	National (9 provinces)	Temperature, Rainfall, Humidity	Waterborne/Enteric	Diarrhoeal disease	Proxy indicator	Anti-diarrhoeal drug sales
Colston et al. (62)	IPD Meta-analysis	SA data subset	Temperature, Rainfall, Soil moisture	Waterborne/Enteric	Enteropathogens (ETEC, rotavirus, etc.)	Laboratory-confirmed (molecular)	Nucleic acid diagnostics
Abrams et al. (52)	Qualitative	Western Cape; Limpopo	Temperature, Rainfall	Waterborne risk context	Exposure pathways	No pathogen outcome	Interviews, surveys
Orievulu et al. (49)	Qualitative	KwaZulu-Natal	Drought	Chronic infection (HIV context)	Treatment adherence	No infection incidence	Interviews
Amegah et al. (43)	Systematic review	SSA (SA subset)	Temperature	Mixed (non-vector borne focus)	Morbidity, mortality	Mixed clinical data	Review synthesis
Chersich et al. (50)	Systematic review	National	Temperature, Rainfall	Mixed	Multiple health outcomes	Mixed	Review synthesis
Godsmark et al. (46)	Case study review	Western Cape	Temperature, Rainfall	Mixed	Policy-level health risks	Not outcome-based	Document review
Khine et al. (48)	Systematic review	National	Temperature, Rainfall	Mixed	Vulnerable populations; pneumonia noted	Mixed	Review synthesis
Wright et al. (47)	Narrative review	National	Temperature, Rainfall	Mixed	Climate-sensitive infections	Narrative	Literature review

The majority of the studies included were to moderate to high quality according to the JBI quality appraisal checklists (see Supplementary File 2). Through a critical analysis of included studies three themes emerged addressing the research question: “What are the emerging trends in EWEs and infectious disease outbreaks in South Africa?” The three themes emerged:

1. “Climate variables”: Climatic impacts on infectious disease highlight how shifts in temperature, humidity, and precipitation influence disease transmission, particularly through waterborne and vector-borne pathogens.
2. “Population vulnerability”: Marginalized communities, including those in informal settlements, face disproportionate health risks due to overcrowding, poor sanitation, and limited healthcare access.
3. “Policy effects”: Impact of EWE's on vulnerability, infrastructure, education and healthcare.

### Climate variables

Increased rainfall and temperature were associated with higher incidence of malaria and diarrhoeal disease in routine surveillance and hospital admission datasets (Table 2). Pneumonia, linked to air-borne viruses also emerged as a sigificant concern (Table 2). Moreover, drought (exceptionally low rainfall) and flooding (exceptionally high rainfall) with co-occurring high temperatures also pose a significant threat to infectious disease outbreaks in South Africa (44, 45). However, other evidence has identified links between rising temperatures, EWE's such as flooding, heavy rainfall, drought, the El Niño Southern Oscillation, La Niña, and the incidence of waterborne and enteric infections including hepatitis, rotavirus, norovirus, enterovirus, cholera, giardia, typhoid, and Legionnaires' disease, which should be taken into consideration (46).

**Table 2:** Synthesized relationships between climate variables and infectious disease outcomes identified in included studies.

**Transmission mode**	**Infectious outcome**	**Level of outcome ascertainment**	**Associated climate drivers**	**Nature of association**	**References**
Waterborne/Enteric	Diarrhoeal disease (non-specific)	Clinical admissions/surveillance	↑ Temperature; ↑ Rainfall; Drought conditions	Increased incidence in children < 5; lagged associations; inconsistent temperature effects in older age groups	(45, 51, 61)
Waterborne/Enteric	Bacterial enteropathogens (e.g., ETEC and other diarrhoeagenic *E. coli*)	Laboratory-confirmed (molecular diagnostics; specimen data)	↑ Temperature; ↑ Soil moisture; ↑ Rainfall	Increased infection risk with higher temperature and soil moisture; rainfall associations pathogen-specific	(62, 65)
Waterborne/Enteric	Diarrhoeal disease (modeled proxy outcome)	Pharmaceutical proxy (anti-diarrhoeal drug sales)	↑ Precipitation; ↑ Humidity; ↑ Temperature; ↑ Evaporation	Climate variables predictive of diarrhoeal burden using ML models; pathogen-specific attribution not available	(63)
Vector-borne	Malaria	Clinical incidence/surveillance data	↑ Temperature; ↑ Rainfall; ENSO-related variability (La Niña)	Increased incidence following co-occurring high temperature and rainfall with ~30-day lag; regional climate influences noted	(44, 45)
Respiratory (environmentally mediated)	Pneumonia (non-pathogen specific)	Clinical admissions	Rainfall variability; Flood-related overcrowding; Temperature variability	Associations with co-occurring rainfall and heat; mechanisms likely indirect (crowding, air quality)	(45, 47)
Mixed/Contextual (review-level synthesis)	Climate-sensitive infectious diseases (multiple categories)	Narrative and systematic review evidence	Extreme rainfall; Drought; Heat stress; Long-term warming trends	Identifies potential amplification of waterborne and vector-borne transmission; highlights data gaps and surveillance limitations	(43, 46–48, 50)

Key ^↑^ = increase.

#### Waterborne and enteric outcomes

Most included studies examining diarrhoeal disease relied on clinically defined hospital admissions or routine surveillance data rather than laboratory-confirmed pathogen diagnoses (43, 45; (61)). Increased rainfall and higher temperatures were associated with elevated diarrhoeal admissions, particularly among children under five years. However, temperature effects were inconsistent in older age groups.

Laboratory-confirmed pathogen data were limited to two studies (43; (62)). These identified associations between higher temperature, increased soil moisture, and rainfall and increased risk of bacterial enteropathogens, including enterotoxigenic *Escherichia coli*. Viral pathogens such as rotavirus demonstrated differing temperature sensitivities.

#### Machine learning and disease prediction

One study applied machine learning techniques to model diarrhoeal burden using anti-diarrhoeal pharmaceutical sales as a proxy indicator across nine provinces (63). Precipitation, humidity, evaporation and temperature were identified as the most influential predictors. However, absence of laboratory-confirmed pathogen data limited pathogen-specific attribution.

A separate individual participant data meta-analysis matched molecularly confirmed enteropathogen infection status with Earth observation-derived hydrometeorological variables (62). Higher temperatures were associated with increased risk of several bacterial pathogens, while viral pathogens demonstrated differing temperature sensitivity. Soil moisture was consistently associated with increased enteropathogen detection.

Both studies reported lag effects between climatic exposure and infectious disease outcomes.

#### Vector-borne outcomes

Vector-borne diseases in South Africa were primarily represented by malaria and schistosomiasis. Malaria incidence was examined using routine surveillance and hospital admission data (44, 45). Co-occurring high temperatures and rainfall were consistently associated with increased malaria incidence, typically following lag periods of approximately one month. Large-scale climatic phenomena, such as La Niña, were also linked to seasonal surges in malaria cases. Schistosomiasis transmission, while water-associated, relies on freshwater snail vectors and is influenced by temperature, rainfall, and humidity, which determine snail habitat suitability and parasite development (47). Warmer and wetter conditions are expected to prolong transmission seasons and expand the geographic range of schistosomiasis.

All vector-borne outcomes were primarily inferred from clinical or routine surveillance data rather than laboratory-confirmed pathogen outbreaks. No included study reported outbreak investigation data with microbiological confirmation of vector-borne infections, limiting pathogen-specific attribution. These findings highlight the sensitivity of vector-borne infections to both short-term EWEs and longer-term climate variability, and underscore the need for integrated climate–health surveillance systems to inform early warning and intervention strategies.

#### Respiratory outcomes

Respiratory outcomes were primarily represented by pneumonia and other acute respiratory infections, assessed using hospital admission records and narrative review evidence (45, 47). Outcomes were clinically defined rather than pathogen-specific. Extreme rainfall events, flooding, and subsequent overcrowding were identified as indirect drivers of transmission, while seasonal temperature variations influenced susceptibility to respiratory infections. Evidence also suggested that air quality, particularly during heatwaves or periods of stagnant atmospheric conditions, contributed to respiratory morbidity.

### Population vulnerability

The health impacts of EWEs are linked to vulnerabilities; human behavioral and socio-economic factors, with those who are more vulnerable more likely to experience disease (48). The occurrence of EWEs intensifies the existing challenges experienced by the population with chronic conditions and adherence to disease management (49). The links between climatic variation and disease are increasingly recognized, with vulnerable populations identified as a priority for national and local government action in relation to EWEs (46).

Due to the high level of underlying morbidity, financial insecurity, poor-quality housing and sanitation, and unreliable access to healthcare, the level of vulnerability in the South African population is high (47). The populations most at risk in South Africa are those over the age of 65 years, pregnant women and the developing fetus, children under 5 years of age, those who work in the outdoor service sectors (such as miners, farmers and other outdoor workers), those with pre-existing medical conditions, those living in poverty, displaced populations and those living in informal settlements (46, 47, 50). Vulnerablity and susceptibility is evident among sub-populations, such as women experiencing higher levels of poverty. The indirect health effects of EWEs is compounded due to systemic discrimination, displacement, unequal access to resources, and unequal roles and responsibilities among genders (46, 48).

Evidence examining a systems approach identified a complex, multidimensional pathway, linked with vulnerability and climatic variables, such as drought and chronic disease management (e.g., HIV), impact on treatment adherence. Also results indicate that drought-induced water shortages (causing reduced access to water and food, loss of livestock and reduced agricultural production) led to disruptions to livelihoods, income, food systems, and general health (49). In addition, during periods of heat stress, access to healthcare and medication is impacted, leading to non-adherence to prescribed treatment and an associated long-term disease management. In breastfeeding women, the risk of mother-to-child transmission increases as hot weather leads to higher intake of HIV-containing breast milk by the infant (47). Furthermore, the impact of social displacement and disruption, alongside gender disparities, in undermining both the prevention and treatment of HIV (50).

Local living conditions and environmental health factors overshadow typical climate-related patterns influencing diarrheal diseases (51). There is increased transmission of infectious disease due to overcrowding, poor sanitation and inadequate access to clean drinking water (46). Continuous access to clean water is a critical factor in reducing vulnerability to infectious diseases. Examination of two distinct regions of South Africa to barriers and vulnerabilities with regards to water, sanitation and hygiene (WaSH), identified that the WaSH services in rural and small towns being influenced by a range of factors, including history (inequalities remain from the Apartheid era), natural environment, socio-economic and infrastructure challenges (52).

### Policy effects

Inadequate transport infrastructure can impact access to healthcare facilities following EWEs. Combined with damage to and flooding of roads, access to medicines and other vital supplies becomes challenging (45). Evidence suggests that national and local government should adopt a “health and climate change in all policies” approach to ensure climate resilience is embedded across multiple sectors, including health, infrastructure, and disaster response (46).

A condensed mapping of key findings to governance domains is presented in Table 3, illustrating how observed climate-sensitive infectious disease risks align with sector-specific policy levers:

- **Water governance and WASH infrastructure functionality** – e.g., water quality, availability, and sanitation system reliability.
- **Informal settlement upgrading and housing resilience** – e.g., flood-proofing, drainage, and resilient housing infrastructure.
- **Primary healthcare reporting and surveillance integration** – e.g., real-time disease reporting, integration of climate and health datasets.
- **Laboratory diagnostics capacity** – e.g., pathogen-specific testing, wastewater surveillance, and environmental microbiology.
- **Risk communication systems** – e.g., public health messaging, early warning alerts, and community engagement.

**Table 3:** Governance domains to mitigate climate-sensitive infectious disease risks.

**Governance domain**	**Key findings from review**	**Relevant policy/action levers**
Water Governance and WASH Infrastructure	Water scarcity, contamination, and inadequate sanitation drive waterborne disease; functionality of water distribution systems is variable; cultural practices affect water use and hygiene (51, 52)	Upgrade water distribution systems; enforce safe water connections; maintain functional sanitation infrastructure; embed climate-sensitive WASH standards into local planning
Informal settlement upgrading and housing resilience	Overcrowding and poor-quality housing exacerbate vulnerability to flooding, heat stress, and vector-borne diseases (46, 50)	Flood-proof and climate-resilient housing; improve drainage and stormwater management; integrate housing upgrades with local disaster risk reduction plans
Primary healthcare reporting and surveillance integration	Incomplete disease reporting, lack of integration with climate and environmental data, limited early warning capacity (46; (63))	Strengthen integration of routine health and climate data; improve timeliness and completeness of reporting; define reproducible surveillance indicators; develop context-specific early warning systems
Laboratory diagnostics capacity	Limited pathogen-specific testing; scarce environmental and wastewater surveillance; constrained capacity for microbiological confirmation (62, 65)	Expand laboratory and environmental sampling capacity; integrate pathogen-specific diagnostics in hospitals; sustain wastewater surveillance; link microbiological data with EWE events
Risk communication systems	Public awareness and behavioral practices influence exposure and risk; limited climate-health communication in communities (47, 52)	Develop targeted risk communication strategies; provide climate-health guidance to vulnerable populations; integrate messaging into disaster response plans

## Discussion

This systematic review set out to examine emerging trends in EWEs and infectious disease outbreaks in South Africa. Overall, evidence indicates a general increase in infection disease irrespective of climatic event type (flood or drought). However, the absence of robust environmental and epidemiological datasets constrains understanding of the specific microbial contributions during EWEs, largely because most studies relied on clinical admissions, routine surveillance, or proxy indicators (e.g., anti-diarrhoeal drug sales) rather than laboratory-confirmed pathogen identification (44, 45, 51, 61, 63). Consequently, while associations between climatic factors (temperature, rainfall, humidity) and disease incidence are observed, causal pathways linking specific pathogens to EWEs remain largely inferential.

Three overarching themes were identified: climatic variables, population vulnerability, and policy effects. EWE's in South Africa are expected to become more frequent and severe, necessitating a stronger integration of health considerations into climate adaptation and mitigation strategies that encompass infectious disease in addition to other multidimensional risk factors (50). Climatic factors such as temperature, humidity, and precipitation were associated with altered transmission dynamics, particularly for waterborne and vector-borne pathogens. Flooding events are often associated with contamination of water supplies, which can increase the risk of waterborne diseases such as cholera and leptospirosis. In addition, stagnant water following periods of heavy rainfall may create favorable conditions for mosquito breeding, potentially elevating the risk of malaria and dengue transmission. However, the reviewed studies generally did not directly measure microbial mobilization or environmental pathogen dynamics, so these mechanistic pathways remain plausible hypotheses rather than empirically demonstrated phenomena. Overall empirical evidence on pathogen distribution in the environment and clinical confirmation during and post-EWE remain limited, representing a critical gap in pathogen surveillance.

Structural barriers within the health system may constrain effective climate-sensitive infectious disease surveillance. Limited laboratory capacity remains a critical bottleneck, particularly in rural and peri-urban districts where microbiological confirmation is not routinely performed for syndromic presentations such as diarrhoeal disease. As noted by Abdullahi et al. (63), stool samples are infrequently tested to determine specific pathogens, restricting the ability to quantify pathogen-specific burdens or attribute outbreaks to climatic exposures. This reliance on syndromic management, while pragmatic in resource-limited settings, reduces the granularity of surveillance data and limits opportunities for modeling climate–pathogen relationships.

Inconsistent reporting from primary healthcare facilities further compounds these challenges. Variability in data completeness, timeliness, and integration with environmental datasets constrains early warning capacity and outbreak detection (47). Fragmentation between meteorological services, environmental health monitoring, and routine health information systems limits the operationalisation of integrated climate–health surveillance. Previous reviews have similarly highlighted the scarcity of empirical health service datasets suitable for robust climate–health analyses in sub-Saharan Africa (50).

Resource constraints, including shortages of trained laboratory personnel, limited diagnostic tests, and competing service delivery priorities, also affect the feasibility of sustained microbiological confirmation and longitudinal pathogen surveillance. These structural limitations suggest that strengthening climate-health preparedness requires both optimisation of existing routine data systems and strategic investment in laboratory diagnostics and environmental sampling capacity. Without addressing these systemic constraints, efforts to develop early warning systems and reproducible surveillance benchmarks may remain limited in scope.

Evidence from seroprevalence meta-analyses demonstrates how population-level biomarkers can serve as reproducible benchmarks to monitor exposure trends (53). Analogous pathogen-specific environmental and clinical benchmarks would further support interpretation of trends following EWEs and improve early warning system functionality.

Population-level vulnerabilities further amplify health risks (46, 47, 50). Marginalized communities residing in informal settlements experience disproportionate burdens due to overcrowding, inadequate sanitation, and restricted healthcare access (52). Displacement following EWEs increases exposure to respiratory and gastrointestinal infections. To reduce vulnerabilities and enhance resilience, results suggest expanding community-based health and social services, particularly in rural and marginalized communities,which are disproportionately affected by EWEs (48). These findings parallel those from high-income settings, underscoring the global relevance of socio-economic disparities in shaping climate-related health outcomes (42).

Infrastructure damage by EWEs compounds these vulnerabilities and intensifies public health crises and delays recovery. Behavioral changes are critical in reducing disease transmission during EWE's, for instance, water storage practices during drought conditions significantly impact the risk of diarrheal diseases, increased water storage, often necessary due to water scarcity and drought, can inadvertently lead to higher contamination risks, contributing to increased diarrhea rates (51). Strengthening climate-resilient health systems, enhancing emergency preparedness, and implementing targeted interventions are therefore essential. In this context, wastewater monitoring emerges as a critical tool: it provides early warning of pathogen circulation, enables rapid detection of outbreaks, and offers insight into microbial dynamics during and after EWEs. Integrating wastewater surveillance into national health strategies would enhance resilience and complement traditional epidemiological approaches (54–56), with South Africa having an established national programme (64).

Emerging technologies offer opportunities to mitigate risks. AI-driven predictive tools, including climate–health early warning systems, show potential for improving outbreak forecasting; however, many existing models rely on proxy indicators rather than confirmed pathogen data, limiting their predictive certainty and generalisability. Moreover, experiences from post-infection sequelae, such as prolonged exertional dyspnoea following COVID-19, illustrate that major health shocks can generate sustained healthcare demand and functional burden (57). This underscores the need for planning that goes beyond acute disease spikes to anticipate medium-term increases in service utilization, chronic care needs, and system strain following EWEs. While studies suggest that incorporating broader environmental variables, such as sea surface temperature and regional precipitation, may improve malaria prediction beyond land-based weather measures, the absence of pathogen-specific validation and context-specific calibration remains a key limitation (44, 58) socio-ecological factors, expanding surveillance capacity, including wastewater monitoring, and embedding climate-health training into medical curricula will be critical for resilience (50, 59). From this analysis strong health-sector leadership is needed to position climate change as a public health issue, alongside community-based approaches that address vulnerability and improve monitoring of pathogen exposure during recovery from EWEs (42).

The emerging evidence suggests there is a need to reframe climate change as a health issue in South Africa, and that healthcare professionals have the potential to be key players in identifying and recording the impacts of climate change on health, and supporting mitigation strategies (50). This systematic review highlights the complex relationships between EWEs, climate variables, and infectious disease outcomes in South Africa. Evidence suggests that extreme rainfall, drought, and temperature variations may influence disease patterns, particularly for waterborne, vector-borne, and respiratory infections. However, due to the predominance of clinical and proxy outcomes, heterogeneity of study designs, and limited pathogen-specific data, these findings should be interpreted cautiously.

Mechanistic explanations, such as microbial mobilization during EWEs, are plausible but not directly demonstrated in the included studies. Strengthening surveillance systems to incorporate laboratory-confirmed pathogens, environmental sampling, and wastewater monitoring will be essential to improve attribution and guide targeted interventions.

Finally, adaptation strategies must consider local socio-economic and environmental contexts, integrating health, infrastructure, and policy measures to enhance community resilience. Investment in climate-resilient healthcare, early warning systems, and pathogen-specific monitoring will be critical to mitigate the public health impacts of increasingly frequent and severe EWEs in South Africa (3, 18). This highlights the importance of considering a “One Health” approach to climate-driven infectious diseases (60). Leveraging technology could enable proactive, context-specific public health strategies to be inacted.

### Limitations and future work

The authors acknowledge that the heterogeneity of study designs, interpretations of EWEs, reliance on proxy outcomes, and lack of outbreak data present a limitation in this analysis. Exclusion of gray literature is also a limitation, as relevant governmental and NGO reports have not been captured. Furthermore, additional data may be available within public health records or unpublished environmental monitoring. However, awaiting permissions and analyzing unpublished data was beyond the scope of this review, and this should be considered a limitation.

### Recommendations for research, policy and practice

#### Actions achievable using existing routine health information systems and climate datasets

- Strengthen data integration: Link routine health facility reporting with local and regional climate datasets to improve monitoring of climate-sensitive infectious diseases.
- Improve timeliness of reporting: Enhance real-time reporting of hospital admissions and surveillance indicators to detect potential outbreaks rapidly.
- Define reproducible surveillance indicators: Establish pathogen-agnostic and pathogen-specific indicators that can serve as consistent benchmarks over time. Experiences from seroprevalence studies illustrate how reproducible population-level benchmarks can provide robust trend interpretation.
- Leverage predictive modeling using existing datasets: Integrate routinely collected climate and epidemiological data into AI-driven early warning systems to anticipate disease surges, while acknowledging that predictive certainty remains limited without pathogen-specific validation.

#### Measures requiring expanded laboratory and environmental sampling capacity

- Sustained wastewater and environmental surveillance: Implement continuous monitoring to capture pathogen-specific dynamics in human and environmental reservoirs during and after EWEs.
- Longitudinal microbiological datasets: Collect detailed laboratory-confirmed pathogen data from clinical and environmental samples to improve understanding of microbial mobilization, seasonality, and outbreak drivers.
- Integrate environmental surveillance with outbreak data: Systematically link microbiological data with patient and community-level exposure to flooding, drought, or other climatic events to refine predictive models and early warning systems.

#### Contextualizing preparedness and medium-term planning

- International experience highlights the importance of reproducible surveillance benchmarks and medium-term health system planning to anticipate sustained burdens following major shocks. In South Africa, this is particularly relevant for climate-sensitive infections where EWEs may cause both acute outbreaks and ongoing demand for healthcare services.
- Policy and planning should consider both immediate outbreak response and medium-term system capacity, including recovery of routine services and management of chronic post-infection sequelae.

#### Strategic priorities

- Integrated pathogen detection and climate-based early warning systems: Combine environmental surveillance, pathogen-specific diagnostics, and predictive models to allow timely identification and response to disease risks.
- Investment in public health infrastructure: Enhance laboratory capacity, water, sanitation, and hygiene systems, and data management tools to support both routine monitoring and crisis response.
- Capacity building: Provide training for healthcare workers in climate-health monitoring, pathogen detection, and early warning interpretation.

Together, these measures provide a pragmatic roadmap for strengthening South Africa's resilience to climate-sensitive infectious diseases, balancing immediate improvements with long-term system capacity and evidence-based planning.

## Data Availability

The original contributions presented in the study are included in the article/Supplementary material, further inquiries can be directed to the corresponding author.
