# Supplementary material for: A systematic review investigating emerging trends between Extreme Weather Events (EWEs) and infectious disease outbreaks in South Africa
Source: Front Public Health. 2026 Mar 16;14:1778784. doi: 10.3389/fpubh.2026.1778784 (PMC13033782; doi:10.3389/fpubh.2026.1778784)
Supplement: Supplementary file 1 [file Supplementary_file_1.docx]

# A systematic review investigating emerging trends between Extreme Weather Events (EWEs) and infectious disease outbreaks in South Africa

Natalie Dickinson^a^, Llinos Haf Spencer^b,c^, Caroline Miller^a^, Nisha Nadesanreddy^d^, Serestina Viriri^e^, Muhammad Zeeshan Shakir^f^, Michael Gebreslasie^g^, David Ndzi^h^, Ozayr Haroon Mahomed^d^, Saloshni Naidoo^d^, Mary Lynch^b^, Fiona L. Henriquez^i*^

^a^ School of Health and Life Sciences, University of the West of Scotland, Paisley, UK

^b^ Faculty of Nursing and Midwifery, Royal College of Surgeons in Ireland, Dublin, Ireland

^c^ Faculty of Life Sciences and Education, University of South Wales, Cardiff, UK

^d^ Discipline of Public Health, Howard College, University of Kwazulu-Natal, Durban, South Africa

^e^School of Mathematics, Statistics, and Computer Science, University of Kwazulu-Natal, Durban, South Africa

^f^School of Computing, Engineering and physical Sciences, University of the West of Scotland, Paisley, UK

^g^School of Mathematics, Statistics, and Computer Science, University of Kwazulu-Natal, Durban, South Africa

^h^School of Electrical and Mechanical Engineering, University of Portsmouth, UK

^i^Department of Civil and Environmental Engineering, University of Strathclyde, Glasgow, UK

***Corresponding author:** Professor Fiona L. Henriquez, Department of Civil and Environmental Engineering, University of Strathclyde, Glasgow, G1 1XJ, UK email: [fiona.henriquez@strath.ac.uk](mailto:fiona.henriquez@strath.ac.uk)

**NIHR WEATHER SR: Search Strategy**

**Search Date:** 11/06/2024

**Dates searched:** Jan 2014 to June 2024

**EBSCO Platform (12 databases) -** **208** (automatic deduplication - 162 into Endnote)

(All databases selected minus eBook collection, PsychBooks, Regional Business News, eBook Open Access Collection and Sage - Transforming Nursing Practice (eBook Sub).)

Abstract search selected.

AB "South Africa" AND ("Extreme Weather Events" OR "Meteorological conditions" OR "Climate Change" OR "Global Warming" OR "Natural Disasters" OR Flood* OR Storms OR "Adverse weather" OR "Climate events" ) AND ( “New conditions” OR “emerging conditions” OR “Emerging trends” OR disease OR Illness OR “disease risk” OR health OR “infectious disease” OR outbreak OR gastrointestinal OR water-borne OR waterborne OR pathogen* OR bacteria OR virus OR amoeb* OR parasit* OR vector OR “E. coli” OR Pseudomonas OR “Vibrio cholerae” OR cholera OR Acanthamoeba OR Giardia OR Schistosomiasis OR cryptosporidium OR Contamination OR "Environmental exposure" OR Malaria)

**Web of Science - 474**

**Topic search selected.**

**((TS=(“South Africa”)) AND TS=(“New conditions” OR “emerging conditions” OR “Emerging trends” OR “disease” OR “Illness” OR “disease risk” OR "health" OR “infectious disease” OR “outbreak” OR “gastrointestinal” OR “water-borne” OR "waterborne" OR “pathogen*” OR “bacteria” OR “virus” OR “amoeb*” OR “parasit*” OR “vector” OR “E. coli” OR “Pseudomonas” OR “Vibrio cholerae” OR “cholera” OR “Acanthamoeba” OR “Giardia” OR “Schistosomiasis” OR “cryptosporidium” OR "Contamination" OR "Environmental exposure" OR “Malaria” )) AND TS=("Extreme Weather Events" OR "Meteorological conditions" OR "Climate Change" OR "Global Warming" OR "Natural Disasters" OR "Flood*" OR "Storms" OR "Adverse weather" OR "Climate events" )**

**Science direct - 366**

‘Title, abstract or author-specified keywords’ selected.

Filtered to research papers and review papers.

Search terms adjusted as only 8 Boolean operators allowed:

**“South Africa”** in ‘find articles with these terms’ **and (Disease OR “human health” OR “infectious disease” OR outbreak OR pathogen) AND ("Extreme Weather Events" OR "Climate Change")**

**Cochrane – 8 (trials)**

Reviews and trials selected.

**"**South Africa" in Title Abstract Keyword AND "Extreme Weather Events" OR "Meteorological conditions" OR "Climate Change" OR "Global Warming" OR "Natural Disasters" OR Flood* OR Storms OR "Adverse weather" OR "Climate events" in Title Abstract Keyword AND “New conditions” OR “emerging conditions” OR “Emerging trends” OR disease OR Illness OR “disease risk” OR health OR “infectious disease” OR outbreak OR gastrointestinal OR “water-borne” OR waterborne OR pathogen* OR bacteria OR virus OR amoeb* OR parasit* OR vector OR “E. coli” OR Pseudomonas OR “Vibrio cholerae” OR cholera OR Acanthamoeba OR Giardia OR Schistosomiasis OR cryptosporidium OR Contamination OR "Environmental exposure" OR Malaria in Title Abstract Keyword - with Cochrane Library publication date Between Jan 2014 and Jun 11th 2024, in Cochrane Reviews (Word variations have been searched)

**Total results:**

8+208+366+474 = 1056

EBSCO removed 46 duplicates

Endnote removed 75 duplicates

Rayyan removed a further 20 duplicates

Total duplicates = 141

1056 – 141 = **915**

**Filtering:**

**Inclusion criteria:** All papers relating to extreme weather events impact on infectious disease in South Africa, written in the English language from 2014 to the present 2024.

**Exclusion criteria:** papers not related to extreme weather events influence on infectious disease outbreaks in SA.

**Stage 1 Filtering:**

915 papers filtered by title and abstract by two authors - 46 papers remaining.

**Stage 2 Filtering:**

Full text screening by two reviewers, followed by further discussion between four of the authors - 12 papers included for data extraction.

**Hand Searching:**

2 additional papers identified through reference list screening: Ikeda et al. (2019) and Ikeda et al. (2017).

**Final Number of Papers: 14**
