# Supplementary figures and images for: A systematic review investigating emerging trends between Extreme Weather Events (EWEs) and infectious disease outbreaks in South Africa
Source: Front Public Health. 2026 Mar 16;14:1778784. doi: 10.3389/fpubh.2026.1778784 (PMC13033782; doi:10.3389/fpubh.2026.1778784)

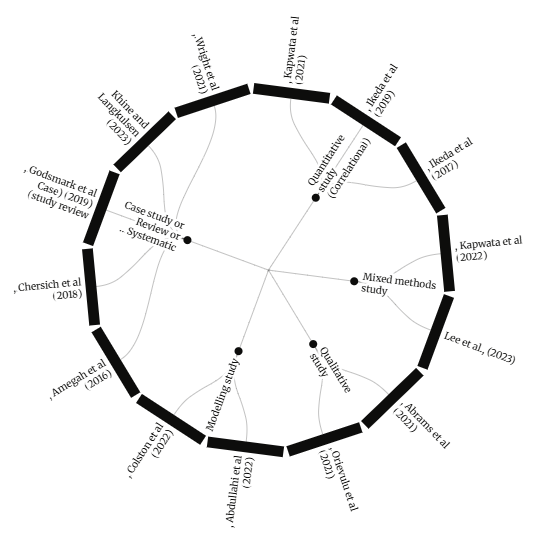


**Figure S1: Overview of types of studies included in this systematic review**

Supplement: Supplementary file 3 [file Supplementary_file_3.docx]
